# Supplementary material for: Iterative improvement in the automatic modular design of robot swarms
Source: PeerJ Comput Sci. 2020 Dec 7;6:e322. doi: 10.7717/peerj-cs.322 (PMC7924708; doi:10.7717/peerj-cs.322)
Supplement: Supplemental Information 3 [file peerj-cs-06-322-s003.zip › argos3/doc/api/standalone/a00489_source.html]

ARGoS: plugins/robots/generic/control\_interface/ci\_colored\_blob\_omnidirectional\_camera\_sensor.h Source File


- Main Page
- Related Pages
- Namespaces
- Classes
- Files

- File List
- File Members

# plugins/robots/generic/control\_interface/ci\_colored\_blob\_omnidirectional\_camera\_sensor.h

Go to the documentation of this file.

```
00001 #ifndef CI_COLORED_BLOB_OMNIDIRECTIONAL_CAMERA_SENSOR_H
00002 #define CI_COLORED_BLOB_OMNIDIRECTIONAL_CAMERA_SENSOR_H
00003 
00004 namespace argos {
00005    class CCI_ColoredBlobOmnidirectionalCameraSensor;
00006 }
00007 
00008 #include <argos3/core/control_interface/ci_sensor.h>
00009 #include <argos3/core/utility/math/angles.h>
00010 #include <argos3/core/utility/datatypes/color.h>
00011 #include <vector>
00012 
00013 #include <argos3/core/utility/logging/argos_log.h>
00014 
00015 namespace argos {
00016 
00026    class CCI_ColoredBlobOmnidirectionalCameraSensor: public CCI_Sensor {
00027 
00028    public:
00029 
00040       struct SBlob {
00041          /* Color */
00042          CColor Color;
00043          /* Angle */
00044          CRadians Angle;
00045          /* Distance in cms */
00046          Real Distance;
00050          SBlob() :
00051             Color(CColor::BLACK),
00052             Distance(0.0f) {
00053          }
00060          SBlob(const CColor& c_color,
00061                const CRadians& c_angle,
00062                Real f_distance) :
00063             Color(c_color),
00064             Angle(c_angle),
00065             Distance(f_distance) {
00066          }
00067 
00068          friend std::ostream& operator<<(std::ostream& c_os, const SBlob& s_blob) {
00069             c_os << "(Color = " << s_blob.Color << "," << "Angle = " << ToDegrees(s_blob.Angle) << ","
00070                  << "Distance = " << s_blob.Distance << ")";
00071             return c_os;
00072          }
00073       };
00074 
00078       typedef std::vector<SBlob*> TBlobList;
00079 
00085       struct SReadings {
00086          TBlobList BlobList;
00087          UInt64 Counter;
00088 
00089          SReadings() :
00090             Counter(0) {
00091          }
00092 
00093          friend std::ostream& operator<<(std::ostream& c_os, const SReadings& s_reading) {
00094             c_os << "Counter: " <<  s_reading.Counter << std::endl;
00095             for (size_t i = 0; i < s_reading.BlobList.size(); i++) {
00096                c_os << "Blob[" << i << "]: " << s_reading.BlobList[i] << std::endl;
00097             }
00098             return c_os;
00099          }
00100       };
00101 
00102    public:
00103 
00107       CCI_ColoredBlobOmnidirectionalCameraSensor() {}
00108 
00112       virtual ~CCI_ColoredBlobOmnidirectionalCameraSensor() {}
00113 
00118       const SReadings& GetReadings() const;
00119 
00123       virtual void Enable() = 0;
00124 
00128       virtual void Disable() = 0;
00129 
00130 #ifdef ARGOS_WITH_LUA
00131       virtual void CreateLuaState(lua_State* pt_lua_state);
00132 
00133       virtual void ReadingsToLuaState(lua_State* pt_lua_state);
00134 #endif
00135 
00136    protected:
00137 
00138       SReadings m_sReadings;
00139 
00140    };
00141 
00142 }
00143 
00144 #endif
```

---

Generated on 10 Jul 2018 for ARGoS by 
 1.6.1
